# From veterinary practice to science: the transformation of dairy cow lameness in Britain, c.1930–1987

**DOI:** 10.3389/fvets.2026.1860297

**Published:** 2026-08-04

**Authors:** Abigail Woods

**Affiliations:** College of Arts Social Sciences and Humanities, University of Lincoln, Lincoln, United Kingdom

**Keywords:** intensive agriculture, clinical veterinary research, dairy cows, lameness, veterinary epidemiology, history

## Abstract

This article offers the first social historical account of lameness in dairy cows, a problem that is widely regarded as one of the most significant threats to their welfare, health and productivity today. Grounded in detailed, critical analysis of original historical documents, it describes the changing manifestations of lameness in Britain from c.1930–1987, as observed, investigated and managed by British veterinarians. It highlights the low inter-war profile of lameness, and how, as dairy farming intensified in the decades after World War II, it was transformed from a sporadic problem of locomotion caused largely by ‘foul in the foot’, into a costly, multi-factorial, herd-level phenomenon associated with diverse foot pathologies. The rising prevalence and complexity of lameness, and its association with new farming practices, was first noticed and investigated by veterinary clinicians, who generated new knowledge of its causes and effects. Although largely forgotten today, their work predated and shaped research performed subsequently by veterinary scientists, who for reasons that will be explained, did not engage significantly with dairy cow lameness until the late 1970s, two decades after clinicians first drew attention to its changing characteristics.

## 1 Introduction

In western countries, over the last 40 years, lameness (along with mastitis and infertility) has been identified repeatedly as one of the most significant threats to dairy cow welfare, health and productivity. It is a painful, multifactorial condition resulting usually from lesions located in the foot, which impair locomotion and reduce the ability to perform natural behavior. Heavy costs are inflicted by reduced milk output, prolonged calving intervals, excessive culling, and the labor required to maintain and restore foot health (1–6). Despite efforts to tackle lameness, prevalence remains high. Around 30% of British dairy cows are believed to be affected at any one time, costing £180 per cow, £15,000 per year for the average sized farm, and £250 m per year for the dairy industry (7, 8). In the USA, prevalence was recently estimated as 10%, and up to 55% in certain regions, costing between $76 to $533 per case (9).

In Britain, dairy cow lameness has not always been such a significant problem. Until the 1950s, it was experienced sporadically, usually in the form of ‘foul in the foot’ (known as ‘foot rot’ in North America), a necrotic condition of the interdigital space that veterinarians usually attributed to bacterial infection (10). Farming and veterinary magazines, journals and textbooks had little to say about lameness, and it had never been subjected to organized scientific research. Over the following decades, however, as production practices intensified in response to a government-led drive to boost domestic food output, lameness was transformed pathologically, epidemiologically and socially into the costly, complex, herd-level threat to welfare, health and productivity that is known as today (11). The purpose of this article is to document and explain its transformation.

Despite the present-day significance of lameness, its history has not been written before [although for an interesting first-hand account, see Sibley (12)]. Like many other endemic problems, it has been sidelined by historians in favor of higher-profile exotic and zoonotic diseases (13, 14). It is important to correct this historiographical distortion. Although endemic diseases typically attracted less political debate, media attention, legislation and economic analysis than exotic and zoonotic diseases, their consistent presence in defined populations or geographic regions had important implications for animals, humans, science and society (15). Recent studies of infertility and mastitis have begun to explore these implications (16, 17). This history of lameness offers a complementary perspective that further illuminates the historical trajectories of today’s most significant threats to dairy cow welfare. It is particularly concerned with how, where and by whom, knowledge of lameness was generated, over the period c.1930–87.

Although recognized from the late 1950s as an increasingly common and costly problem characterized by diverse foot pathologies whose causation was linked to new production practices, the first substantial, publicly funded programme of research into lameness did not begin until 1977. This was many decades after the commencement of research into other prominent threats to dairy cow health and (re)production. Rather than criticizing the scientific ‘neglect’ of lame cows, this article seeks to understand their long absence from veterinary scientific research institutions, while simultaneously drawing attention to other important sites of knowledge-building, specifically veterinary clinical practice. Like their medical counterparts, the general practitioners, veterinary clinicians were frontline responders to endemic diseases, and therefore well-placed to observe and study them (18). While the authority of their field-based learning has been undermined in recent decades by the rise of Evidence-Based Veterinary Medicine (EBVM) (19), this study demonstrates its importance to the history of lameness.

## 2 Materials and methods

Informed by Charles Rosenberg (20), whose research on the history of disease is considered a cornerstone of the social history of medicine, this study approaches dairy cow lameness as both a social and a biological phenomenon, which had a ‘real’ pathological existence in the bovine body to which humans gave meaning [‘In some ways disease does not exist until we have agreed that it does, by perceiving, naming and responding to it’ (20, p. 1–2)]. It not only investigates the changing causes and manifestations of lameness, but also the people, policies, activities, institutions, hierarchies, relationships, identities, interests, values, priorities, discourses and assumptions that shaped how it presented, was perceived and acted upon (21). A similar approach to disease is adopted by social scientists, who explore its construction through particular social relationships, value systems, scientific methods and medical technologies (22, 23). Epidemiologists also endorse this approach because ‘how societies generally recognize, define, name, and categorize disease states and attribute them to a cause or set of causes’ can have profound effects on the state of individual and population health (24).

Following standard historical research methodology (25), the study directed general queries about lameness at surviving source materials, and drew on existing historical literature to help interpret the findings. The aim was to extract meaning and construct an explanatory narrative of change over time. The general queries were: How, in mid-20th century Britain, did society and biology interact to establish lameness as the complex, high-profile threat to dairy cow welfare, health and productivity that it is today? How did lameness change over time and how did veterinarians experience, interpret and respond to these changes? Having determined that veterinary research establishments did not commence research into lameness until the late date of 1977, efforts were made to explain this finding by interrogating their structures and cultures. The starting date of c.1930 was selected to capture perspectives on lameness that predated the use of antibacterial drugs. The end point of 1987 was determined by the closure of the first major lameness research programme. It predates the first (1988) published report of digital dermatitis, which drove further changes to the demographics of lameness in Britain (26).

Due to its extensive reach and rich content, the most important historical source for this investigation was the *Veterinary Record* (*VR*). It was (and still is) a weekly publication read by most British veterinarians and issued by their representative body, the British Veterinary Association (BVA). To counteract inadequacies in digital indexing, its indexes were examined manually and systematically from 1930 to 1987 for references to lameness and its synonyms (e.g., foul, foot lameness). This approach identified not only scientific articles - which after Latour and Woolgar (27), we recognize as crafted to present research as objective and inevitable, and to erase many of its messy, contingent, and negotiated aspects - but also news, correspondence, editorials, and reports of discussions at veterinary meetings. These provided less mediated insights into lameness, and privileged the experiences and opinions of veterinary clinicians. In revealing the individuals and institutions that shaped the history of lameness, analysis of the *Veterinary Record* stimulated a further search for other relevant source materials, including institutions’ annual reports, the biographies of key individuals, their other lameness publications, and the articles that they cited. Sources which were not available online were obtained via the British Library. All materials were read partly for content – to understand how lameness presented, was understood and managed – and also, importantly, for context: to probe the identity, background and motivations of the author; the experiences, knowledges and methods they drew upon, and for whom they were writing. Throughout, efforts were made to identify and account for absences in the historical record by asking who was *not* writing about lameness, what was not being said and done about it, and why.

Forms of lameness are referred to using terminology that was current at the time, with contemporary terms added, where appropriate, to aid comprehension by veterinary readers. For consistency, the term ‘veterinary clinician’ is used (in preference to ‘veterinary practitioner’, or ‘practicing vet’) to refer to veterinarians who were primarily engaged in providing clinical care to animals. Clinicians usually worked in private veterinary practice, with roles in university veterinary departments opening up toward the end of our period. The term ‘veterinary scientist’ is reserved specifically for individuals whose primary role was to scientifically investigate animal diseases. Most worked in dedicated research laboratories supported by the publicly-funded Agricultural Research Council (ARC), Ministry of Agriculture, Fisheries and Food (MAFF), private pharmaceutical companies and the charitable Animal Health Trust. While some university-based veterinary staff also engaged in scientific research, they were classified as veterinary teachers in official reports (28). For this reason, and because the period covered by the article predates the mainstream recognition and institutionalization of clinical research, it uses the inclusive term ‘knowledge-building’ in preference to ‘research’, to refer to activities through which new understandings of lameness were generated.

## 3 Results

The results of this analysis are summarized in Table 1 and elaborated chronologically in the text below.

**Table 1:** Lameness history: a chronological and conceptual framework.

Dimension	c.1930–1955	c.1955–1968	c.1968–1977	c.1977–1987
Lameness status	Low-profile, private problem	Increasingly significant disease	Complex, costly, herd-level problem	Major national threat to welfare, health and productivity
Lameness characteristics	Sporadic individual cases Predominantly foul in the foot	Rising incidence, growing diversity of lesions Early links to changing production systems	Rising incidence, diverse pathologies Multifactorial, herd-level disease attributed to production practices	Statistical insights layered on existing understandings Incidence of different pathologies and relative contributions of causative factors are quantitatively defined
Knowledge production	Limited, individual, experiential Informed by lameness in horses	Growth of experiential knowledge Practice-based pathological enquiries Early synthesis (e.g., theses, articles)	Larger-scale, systematic, clinical enquiries at university field stations Textbook synthesis (e.g., *Lameness in Cattle*, 1972)	Led by veterinary scientists with clinical collaborators Grounded in analysis of large-scale, practice-based surveys Scientific outputs and clinical communications
Drivers of change	New sulphonamide treatment for foul	Post-WWII agricultural production drive New breeding, feeding, and husbandry practices Increased veterinary attendance on farms	Continued drive to increase productive efficiency More intensive systems that increase lameness vulnerabilities Appointment of clinical veterinary lecturers	Rising awareness of lameness complexity and implications for welfare and productivity Growing emphasis on disease prevention New epidemiological methods and computing technology New research funding regime.

### 3.1 The identity and management of lameness c.1930–55

Farmers in inter-war Britain were accustomed to seeing their dairy cows fall lame. Except in cases of accident or injury, they usually attributed lameness to foul-in-the-foot (equivalent to ‘foot rot’ in North America), a loosely defined entity that included the disease known today as interdigital necrobacillosis. Veterinarians implicated the bacterium *Bacillus* necrophorus (since renamed *Fusobacterium necrophorum*). Found in feces and soil, it was thought to gain entry to the foot after injury, bruising from hard surfaces, or softening of the horn under wet conditions (29). According to Black’s Veterinary Dictionary (which was a ‘go to’ veterinary reference work that went through multiple editions), foul was normally sporadic and non-contagious, but could assume a contagious form. The hind feet were particularly affected, and in the worst cases, the limb was completely non-weight-bearing. One characteristic sign was for the cow to ‘suddenly stop walking, and shake the affected foot.’ On inspection, an area of reddened, swollen skin could be seen between the digits, which developed into dead, discharging, foul-smelling tissue that gave the disease its name. In serious cases, infection could penetrate deep into the tissues, tendons and joints, causing foot swelling and deformity (30, p. 394–5).

Farmers typically responded by cleansing, poulticing and dressing the foot (30). Accustomed to self-help, and suffering economically from the prevailing agricultural depression, they sought veterinary assistance only in severe or unresponsive cases, or for highly valuable cows (16). Veterinary clinicians adopted a surgical approach to foul. After immobilizing the cow, they used a sharp hoof knife to remove damaged horn and tissue, open up abscesses, and clean out discharging sinuses before applying antiseptic dressings (31). This type of reactive, manual response to individual sick cows was typical of veterinary farm practice at the time and was sometimes referred to as ‘fire brigade’ medicine (16). While foul was not fatal, it frequently resisted treatment, and symptoms could persist for months (29, 31, 32).

Soon after World War II, a new pharmaceutical treatment emerged: intravenous sulphonamide. Early veterinary adopters reported that symptoms quickly resolved (31, 32). Unable to administer the drug themselves, farmers sought veterinary aid more frequently. One veterinary clinician reported in 1949 that “It is now rare for a day to pass without one of our clients wanting a cow ‘injected for foul’” (33, p. 611). A 1951 analysis of case records drawn from three ‘representative’ veterinary practices showed that lameness comprised 2.81% of all conditions treated. Although significantly below mastitis (14.75% of conditions treated), and infertility (6.3%, primarily due to abortion and unknown causes), it ranked as one of the commonest causes of veterinary visits to farms. With a total of 271 cows treated in 231 initial visits, it was evidently a problem of individual cows rather than the herd (34).

Despite its growing importance to veterinary clinical work, lameness remained a low-profile problem until the mid-1950s, rarely featuring in articles, journal correspondence, or veterinary meetings. Except for a new reference to sulphonamide treatment, entries for ‘foul in the foot’ remained virtually unchanged in six successive editions of Black’s Veterinary Dictionary (1928–62), pointing to the static nature of veterinary knowledge. No research was conducted. This cannot be attributed to a lack of scientific resources because although the interwar veterinary scientific establishment was small, it was active, particularly in dedicated institutions like the MAFF Veterinary Laboratory, and the ARC’s new field station at Compton, Berkshire. Staffing and facilities expanded after World War II to support the national drive for food production (35–37). By 1954, there were 224 full-time veterinary scientists (not including university staff) out of a total veterinary population of 4,241 (28). They continued to ignore lame cows.

This situation occurred partly because lameness lacked mystery and urgency. It was a sporadic and easily diagnosed disease, caused usually by foul - which could be managed, albeit reactively and with no guaranteed cure. Consequently, clinicians rarely needed to seek support from the publicly-funded Veterinary Investigation Centers, whose interventions drew attention to prevalent or puzzling diseases that merited scientific investigation (35). The common belief that lameness in dairy cows was analogous to lameness in horses also helps to account for the lack of research performed on it, because it meant that as in horses, lameness was defined as a problem of locomotion (30, 38).

Although declining in importance, horses exerted an outsized influence over veterinary training, practice and professional identity (39). As illustrated by the old adage, ‘no foot, no horse’, their prime function was to provide draught power. This function was disrupted most frequently and significantly by lameness, which therefore prompted frequent veterinary attendance and a raft of veterinary literature (40). Apart from having two digits, cow’s feet were believed ‘in other respects constructed on the same general principles as that of the horse’ (41, p. 255), such that ‘nearly all the diseases of the feet encountered in the horse are met with in the ox’ (42, p. 1040). [Their resemblance to sheep’s feet, which were also very prone to lameness but rarely attended by veterinarians, was not commented on (43)]. Foul in cattle was said to resemble ‘quittor’ in the horse (both could manifest as localized soft tissue swellings at the coronary band that burst to produce suppuration). Other forms of lameness were referred to using equine lameness terminology, for example ‘sandcrack’ (a fissure in the wall of the hoof), ‘joint ill’ (abscesses in the joints of young animals caused by infection entering via the navel) and laminitis (painful inflammation of the sensitive inner tissues of the foot) (41, 42, 44).

However, unlike horses, cows were no longer draught animals (45). They were expected to produce milk and calves that generated profit for farmers and nutritional benefits for consumers. From the 1930s, the enhancement of their productive functions was prioritized under national public health and agricultural policies that aimed to address under-nourishment in poor urban children, increase farming profits during the interwar depression, and feed the nation in wartime (17, 46, 47). Problems like mastitis, infertility and abortion, which had their seats in the cow’s organs of (re)production, and undermined milk and calf production in very visible ways, attracted attention in surveys of dairy cow ‘wastage’ (or premature disposal), veterinary professional and policy discussions, and veterinary scientific research (16, 48). By contrast, lameness, viewed as a problem of locomotion rather than production, was rarely remarked upon outside of the private veterinary clinical encounter.

### 3.2 Lameness transformed: practice-based observations c.1955–68

During the late 1950s, veterinary clinicians’ experiences and perceptions of dairy cow lameness began to shift. They observed that lameness was becoming more common, its pathologies were diversifying, milk production was affected, and sulphonamide was not, after all, a wonder drug. These views featured in, and may have prompted the organisation of, the first ever panel on lameness, at the 1960 annual congress of the British Veterinary Association (BVA) (49, 50). Clinicians were well represented among the speakers and discussants. Colin Wood, who ran a practice in Dumfries, Scotland, and G. H. Arthur of the Royal Veterinary College, London, both described how to manage lameness surgically (44, 51). According to Arthur, this ‘required considerable willpower. It involved a tremendous amount of sheer hard work, often under very unpleasant conditions. It was time-consuming, not particularly remunerative, required adequate lay help, and taxed the ingenuity of the surgeon in matters of restraint and anesthesia’ (51, p. 1223). Observing that lameness caused great suffering, ‘reduction in milk yield and loss of flesh’, he recommended removal of the affected digit in cases of deep-seated infection (51, p. 1215). Paul Greenough opened the discussion with a slide show of foot lesions that he had encountered in his practice, one of the largest in southern England, where lameness accounted for 5% of all clinical work. While acknowledging that foul remained the commonest cause of lameness, he drew attention to other, lesser-known pathologies such as white line disease (where the hoof wall separated from the sole horn, allowing infection to enter) and corkscrew claw (a conformational defect) (52, 53).

All three men criticized scientists’ lack of interest in lameness. Greenough had identified just sixty publications in the international scientific literature over the previous sixty years and only seven by British authors, many relying on the horse as a reference point (52). The men also highlighted factors that contributed to lameness: trauma to hoof horn caused by hard surfaces or sharp stones, exposure to muddy wet conditions which macerated the skin, and overfeeding in the case of laminitis. Clinicians in the audience confirmed and extended these observations. Former BVA president, H. F. Hebeler, remarked that ‘as production, in dairy cows particularly, was improved, so the occurrence of foot diseases increased.’ He pointed to genetic background as a cause of poor leg conformation (54). Other clinicians flagged conditions underfoot, the particular susceptibility of Friesian and Ayrshire cows, and the possibility that bulls used for artificial insemination were to blame (49).

These observations reveal that veterinary clinicians were beginning to associate the apparent deterioration in foot health with changes in cattle production practices. These changes were encouraged by government policies aimed at increasing domestic agricultural output and farming efficiency. They were supported by production subsidies (whose value decreased over time, encouraging the search for efficiency savings), capital grants for new cow sheds and milking parlors, free advisory services, and investment in agricultural and veterinary research. The overall trend was to intensify production (37, 55). Farmers bred cows increasingly by artificial insemination, such that by 1958, a few hundred elite bulls were inseminating 58% of the cows in England and Wales (56). Traditional dairy shorthorn cows gave way to higher-yielding Friesians, fed more intensively on improved pastures, commercial compound feeds and mineral supplements. By 1965, Friesians made up 64.2% of the dairy cows in England and Wales compared to 40.6% a decade previously. The average milk yield per lactation had jumped to 3,545 liters from 2,545 before the war and would increase further to 4,070 liters by 1975. Farmers also moved away from labor-intensive cowsheds where dairy cows were tethered, fed and milked on standings, to loose housing systems with separate milking parlors (37). These systems included straw-bedded yards, cow cubicles (first designed by Cheshire farmer, Howell Evans in 1960, as individual, raised, bedded areas separated by open concrete walkways and dung channels) and houses with slatted floors [which were shown in a 1965 study of farm buildings to cause leg or foot damage in 3% of cows (57, 58)].

These changes impacted not only on foot health, but also on the pathology and epidemiology of other common health problems like mastitis and infertility (17, 59). Veterinary clinicians were well placed to observe the health consequences because rising cow values, greater farming affluence, and the advent of new vaccines, antibiotics and infertility treatments made farmers more inclined to employ them (16). Some, like Greenough, began to actively investigate lameness. In 1961, he submitted a thesis describing diverse pathologies for the prestigious award of Fellowship of the Royal College of Veterinary Surgeons (FRCVS) (60). Later published as an article in the *Veterinary Record* (61), it drew on 10 years of practice-based investigations. Reiterating criticisms of the veterinary tendency to elide lameness in the horse and cow, Greenough attributed the decline in foot health on the ‘grossly abnormal conditions found on some of our farms’, for ‘the domestication of animals can only be successful if they are bred to withstand their new and abnormal environment’ (61, p. 61). In addition to surgical treatments, he instructed stock-owners to ‘pay more attention to the factors predisposing to foot disease’, which he identified as cow genetics, metabolisms, conformation, gait, and conditions underfoot (61).

Greenough’s work appears to have encouraged other veterinary clinicians to publish their experiences of lameness (62–64). Another clinician, Colin Maclean, commenced a seven-year practice-based study of laminitis in cattle for which he, too received the FRCVS (65, 66). Supported by post-mortem inspection in abattoirs, and laboratory analyses of haematology and blood biochemistry, Maclean observed affected cows in four different counties, noting their symptoms, gait, course of illness and foot pathology, and exploring associations with age, diet, heredity, calving and concurrent diseases. He concluded that laminitis was under-diagnosed, potentially hereditary, often associated with other illnesses, and occurred particularly around calving-time.

Despite their increasing numbers (from 224 in 1954 to 530 in 1969), veterinary scientists took little interest in clinicians’ observations of the increased incidence of dairy cow lameness, its diversifying pathologies and association with intensive husbandry practices [although interestingly, sheep lameness was investigated, largely due to efforts by Australian pathologist, Prof WI Beveridge of Cambridge University, who persuaded MAFF veterinary scientists to test out his imported ideas and control methods (43)]. A ‘Diseases of Cattle’ Bulletin issued in 1964 by the MAFF Veterinary Laboratory Service, focused exclusively on foul in the foot as a cause of lameness and deviated from an earlier edition only by reference to disinfectant footbaths and sub-cutaneous injections of sulphonamides (67).

Continuing scientific disinterest in dairy cow lameness occurred alongside the intensification of research into udder and reproductive diseases. The ARC granted its scientists considerable freedom to select their research subjects, in the belief that findings of importance to agriculture were more likely to be achieved from investigations that sprang ‘from the interest and enthusiasm of the scientist’ than from ‘work carefully directed to desired ends’ (68, p. 11). Further work on udder and reproductive diseases appeared justified because their incidence and complexity also appeared to be increasing (17, 60), while the design of disease surveys conducted by MAFF veterinarians obscured the impacts of lameness (69). In the first national survey of dairy cow diseases (1957/58), diseases were divided into three major categories: those associated with parturition (including abortion), infectious diseases (including mastitis) and non-infectious diseases (including ‘undiagnosed infertility’). Confusingly, ‘foul’ featured under infectious diseases, while ‘lameness’ was categorized as non-infectious and then subdivided into lameness associated with injury, arthritis, rheumatism and ‘infection other than foul.’ None of these headings adequately captured the range of foot pathologies identified by Greenough. Had all records been amalgamated, lameness incidence would have totaled 3.88%, placing it among the five most common dairy cow diseases (though still some way behind mastitis, at 10.3%, and infertility (including stillbirth and abortion) at 6.8%). However, authors of the survey report drew attention only to foul, whose incidence was 2.8%. In their commentary, they portrayed it as an outlier among the 11 diseases that had an incidence above 1% because it was the only one not ‘peculiar to the female’ (70, p. 26).

BVA leaders did not dispute the importance of udder and reproductive diseases, and the need to research them. However, they did question whether, at a time when husbandry practices and disease demographics were changing rapidly, researchers were fully engaging with the economically important diseases that clinicians encountered on farms (71, 72). A study conducted by Dr. Jack Payne of the ARC’s Institute for Research on Animal Diseases suggested they were not (73). Payne counted the number of scientific papers published on cattle diseases over the period 1959–63, and compared the findings with the results of the 1957/58 national dairy disease survey. He also asked a random sample of veterinary clinicians to select which five conditions they encountered most frequently. Their responses placed lameness third (it was cited 106 times), just behind mastitis (110) and calf scours (112), and some way ahead of infertility (68, including abortion). Payne concluded that lameness was the most under-researched of all cattle health problems.

During the 1960s, veterinary research institutions did begin to engage more seriously with the health consequences of intensive husbandry practices. They also moved beyond traditional bacteriological methods to explore molecular biochemical, physical and immunological processes and their disruption by disease (74, 75). However, as a biomechanical problem that veterinary clinicians attributed to the interaction between changing cattle conformations and environments, foot lameness was not amenable to these modes of investigation. The ARC did organize a 1964 conference on foot disorders, attended by members of government departments, veterinary schools, and agricultural and commercial organizations. Their observations included the need for a clearer clinical and pathological definition of foul ‘which only further research can provide’ (76, p. 61). However, no such research was commissioned by the ARC.

### 3.3 Lameness transformed: university-based clinical studies, c.1968–77

Despite the lack of veterinary scientific interest, advances in understandings of lameness continued to occur. Veterinary clinicians were again in the driving seat, now working particularly at veterinary school field stations, which were established as part of the post-WWII transition in veterinary education from private schools to university-based degree programmes. Clinicians were appointed to these stations, to instruct students in preventive medicine, livestock husbandry, veterinary medicine and surgery, using clinical cases referred from nearby farms and veterinary practices. There was little expectation that they would conduct research. Veterinary schools received a relatively low proportion of the nation’s veterinary research funding (28, 77), and the concept of clinical veterinary research was only just emerging, primarily in pharmaceutical companies that defined it narrowly as the field testing of veterinary drugs. As late as 1989, clinical veterinary research was described as ‘of modest achievement thus far’ (78). This situation contrasted with human medicine, where clinical research took off in the decades after WWII with the formation of the National Health Service, and the creation of new clinical chairs in medical schools and research units under the Medical Research Council (79, 80). Nevertheless, field station staff did engage with health problems encountered in private veterinary practice, as retired clinician, Pam Owen later recollected:

A disease […] would crop up somewhere. And if you were meeting other veterinary surgeons in a social context, you might just say, ‘I’ve seen a very peculiar animal today and it did this, that and the other.’ And he or she might say, ‘Well, seeing as you say that, I think I saw one last week’ […] and slowly the information would seep through veterinary practices. And then, of course, the university veterinary programmes would get interested (81).

At the University of Liverpool’s veterinary field station, Leahurst, David Prentice and P. A. Neal conducted a highly influential study of lameness using records of seven dairy herds attended by its farm animal practice (82). They reported that over the period 1965–68, a staggering 30 percent of cows experienced lameness each year (nearly a 10-fold increase on the combined 3.88% incidence measured by the 1957/58 national survey). They identified structural abnormalities (not always resulting in clinical lameness) in 76% of the feet they inspected at a local knacker’s yard. They also itemized 18 underlying pathologies and their relative frequencies. Sole ulceration had overtaken foul as the most frequent problem. For unknown reasons, cows were particularly susceptible to lameness in the three months after giving birth. Critically, this was the period in which they yielded the most milk. The authors concluded by emphasizing the economic importance of lameness and the need for further research. The same message was conveyed by Somerset clinician, Roger Eddy, in a published analysis of lameness cases seen by his six-man practice in 1973. Although he reported a different distribution of pathologies, and a much lower incidence than Prentice and Neal, he, too, observed an association with the early part of the lactation cycle (83).

Taking advantage of Leahurst’s access to cows, Prentice went on to investigate ways of improving lameness diagnosis and management, paying particular attention to the claw growth (84). With anesthesia and imaging expert, Gwyn Wyn-Jones, he devised an angiographic technique to help visualize vascular disruption, which was implicated in laminitis and sole ulcer, and a local anesthetic technique for use in foot surgery (85). At the University of Glasgow’s veterinary field station at Garscube, strong links developed between clinicians and pathologists, resulting in a standard approach to clinical cases involving X-rays, laboratory tests, post-mortem examinations, and visits to the animal’s home farm. This system, together with high standards of record keeping, allowed general observations to be drawn about the nature of particular diseases (86, 87). Clinical lecturer David Weaver, took advantage of this system to advance knowledge of lameness. Appointed in 1961, after several years’ experience in private practice, he won a prestigious Alexander von Humboldt Foundation fellowship to visit European veterinary schools with interests in cattle digital diseases, including the Hannover veterinary school (88, 89). The first of his many publications on cattle lameness appeared in 1969 (90), describing the presentation, diagnosis and outcomes of 40 cases of hip lameness attended at Glasgow over a five-year period. Two years later, he reported his investigation into an outbreak of ‘solar penetration’, which he estimated to have cost the farmer £8,000 over two years due to reductions in milk yield (91).

In 1974, Weaver published an extremely detailed account of diseases of the cow’s interdigital space (92), which differentiated conditions formerly collapsed under the heading of ‘foul’ into five different categories. He recommended that vets not only treat affected cows but also give preventive advice: on constructing suitable buildings, avoiding overcrowding, using formaldehyde foot baths to clean and toughen feet, and not breeding from bulls with feet abnormalities. Following Prentice and Neal’s lead, he also used the abattoir as a research resource, to find out why cows’ hind limbs had been condemned as unfit for human consumption. This study drew attention to conditions affecting the upper limb such as osteoarthritis (93).

Meanwhile, in 1966, on the back of his FRCVS study on diseases of the bovine foot, Paul Greenough took up a position in the large animal clinic of the newly established Western College of Veterinary Medicine at the University of Saskatchewan (53). The College’s founding Dean, Larry Smith, had intentionally integrated the basic sciences, pre-clinical science and clinical division, promoting collaboration and cooperation between them (94). One of the outcomes was a collaboration between Greenough and F.J. MacCallum, Professor of Veterinary Anatomy. Together with Glasgow’s David Weaver, they co-authored the first English language textbook on *Lameness in Cattle,* which appeared to great acclaim in 1972 and went through three editions. It was a clinically oriented volume directed at an international audience. The authors justified their study by the ‘appalling losses’ that lameness inflicted, the paucity of scientific research on it, and the fact that ‘in many Western European countries more visits are paid to lame cattle by the veterinarian than to any other form of disease in this species’ (11, p. xiii, 3).

After an introduction that covered the functional anatomy of the limb, methods of restraint, anesthesia, radiography and general examination, the volume documented the different forms of lameness then known to veterinarians, organized by anatomical location (foot and upper limb) and the structures affected. It described their clinical course, pathology, diagnosis, contributing factors, treatment and methods of prevention. Drawing on audio-visual skills that Greenough had applied in talks to veterinary and farming audiences, conditions were illustrated using radiographs, photographs and line drawings. To overcome national differences in nomenclature, the authors employed anatomical terminology: foul became ‘interdigital necrobacillosis’, ‘sandcrack’ - ‘vertical fracture of hoof wall’, and ‘bruised sole’ - ‘pododermatitis circumspecta’ (11). Terminological adjustments were made in the second edition of 1981, following the adoption of approved nomenclature at the first international symposium on abnormal foot conditions in Utrecht, 1976. Weaver, Greenough and Dutch veterinarian, Dr. Egbert Toussaint Raven, drove the organization of this meeting, which established lameness as an international issue, and the symposium as a biennial event, to which veterinary clinicians continue to make significant contributions (95, 96).

The authors of *Lameness in Cattle* described the manual treatment of individual cases of lameness, now facilitated by crush cages for restraining cattle, new methods of local anesthesia, X-ray diagnosis, broad spectrum antibiotics, topical sprays, and special compounds to protect the foot. They also discussed general measures such as foot baths and preventive and corrective foot trimming. In addition, they identified many predisposing factors whose removal could potentially prevent lameness: inheritance (of anatomy and constitution), nutrition, and environment. Conditions underfoot were particularly important. Soft litter encouraged hoof overgrowth and deformity, slippery floors made cows prone to injury, hard concrete surfaces wore down their soles making them vulnerable to punctures and ulcers, long walks from pasture to milking parlors on loose stone roads encouraged objects to penetrate their soles, while muddy areas around drinking troughs became heavily contaminated with infection (11).

The contents of the volume demonstrate that by 1972, lameness in cows was no longer considered analogous to lameness in the horse. The authors viewed it as a problem of production, not locomotion, and broke new ground in attempting to calculate its economic impact. While acknowledging the difficulties - because lameness affected different cows in different ways – they showed that on average, the milk output of a lame cow fell by 20 percent over a nine-month lactation cycle. Together with loss of flesh, premature culling, and the costs of veterinary treatment and cow replacement, losses from lameness in Britain amounted to at least two million pounds per annum.

### 3.4 From practice to science: lameness, 1977–87

In 1977, the first substantial programme of lameness research was launched at the ARC’s Institute of Research into Animal Diseases (IRAD), Compton, Berkshire, in collaboration with David Weaver at the University of Glasgow. Led by a team of statisticians, it continued until 1987 when institutional restructuring forced its termination. It aimed to build knowledge of lameness epidemiology and produce guidelines for prevention (97). It was motivated partly by changing experiences and observations of lameness, as outlined above, and partly by other factors which overcame previous barriers to research and enhanced its urgency.

One such factor was the growing concern for animal welfare (98). In her influential 1964 volume, *Animal Machines*, Ruth Harrison had highlighted the detrimental effects of intensive husbandry systems on livestock welfare (99). It caused the government to appoint a Technical Committee to Enquire into the Welfare of Animals Kept under Intensive Livestock Husbandry Systems, known after its chair as the Brambell Committee. The Brambell Report stated that ‘a principal cause of suffering in animals… is disease’ and observed that keeping dairy cows exclusively on slatted floors caused foot discomfort, which manifested in reduced milk yields (100, p. 45). It stimulated new legislation, under which MAFF issued its first cattle welfare code. This listed lameness as one of several symptoms of ill health (and consequently poor welfare). It emphasized the need to treat affected cows without delay and seek veterinary advice if necessary. It also gave general recommendations on housing for good welfare (101).

A second factor was the government’s renewed emphasis on domestic food production (37). In 1975, responding to economic pressures and volatile global commodity prices caused by the 1973 oil crisis, it released a white paper entitled “Food from Our Own Resources” (102). This signaled the need to boost output and productive efficiency, and targeted milk yields for improvement through advances in health and genetics. In this context, the newly quantified impact of lameness on milk yield identified it as a problem in need of resolution.

Third, while lameness was not amenable to investigation by dominant bacteriological and biochemical research methods, new epidemiological methods offered the prospect of shedding new light on it (103–105). Emerging in human medicine around 1950, and seen in Doll and Hill’s famous studies of lung cancer and smoking, and the Framlingham study of coronary heart disease, they used quantitative analysis to identify a web of disease risk factors, whose management could help to prevent disease. Epidemiological analysis was further encouraged by the adoption and use of electronic computers in the 1960s and 70s (106), and a growing emphasis on disease prevention which reflected rising concerns around antimicrobial resistance. A committee appointed by government to examine these concerns (known after its chair as the Swann Committee) reported in 1969 that livestock disease patterns were changing as husbandry systems intensified, and that to reduce the need for antibiotics, epidemiological research was required to inform ‘purposive, enlightened, and profitable changes in the methods of animal husbandry’ (107, p. 54). Veterinary leaders supported this recommendation because it aligned with, and provided a scientific underpinning for, their longstanding drive to promote ‘preventive veterinary medicine’ in clinical practice. Distinct from the traditional ‘fire-brigade’ management of individual sick animals, it prioritized the delivery of holistic, husbandry-based preventive advice (108).

Fourth, the freedom of ARC-funded scientists to select their own subjects of investigation was curtailed following a 1971 government-commissioned review of ‘The Organization and Management of Government Research and Development.’ Known after its author as the Rothschild report, it argued that publicly funded research should be more responsive to agricultural needs. The government responded by transferring the ARC’s budget to the Ministry of Agriculture, which used it to commission projects aligning with its own priorities (109).

The influence of these various factors can be seen in a *Veterinary Record* editorial published alongside Weaver’s 1974 article on diseases of the cow’s interdigital space. After praising Weaver’s ‘helpful contribution to our knowledge’, the editor declared that ‘prevention must always be our aim’, for ‘lameness means pain to a varying degree, which if acute leads to lowered production.’ Criticizing the low profile habitually awarded to lameness, and its treatment as a ‘particular and local problem’, they highlighted the many gaps in understanding, and called for its recognition by the new research commissioning board, appointed to determine where funds should be expended (110, pp. 113–14).

Finally, in 1977, MAFF directed funding to IRAD for research into dairy cow lameness. Directed since 1973 by Dr. Jack Payne (known for his articulation of the Production Disease concept, and who authored the 1966 article which identified lameness as a significantly under-researched disease), it was a highly appropriate venue, devoted to research on diseases that undermined production (74, 111). Lameness was taken up by its team of statisticians, whose only veterinary member, Dr. Alexander Russell, justified the investigation as follows:

When herds with greater susceptibility than normal encounter environmental conditions which impose stress onto the legs and feet, many cows may become lame, resulting in considerable loss of production and increase in costs. In addition the welfare aspect of the problem cannot be ignored. The relative incidence of the many different lesions were not known, and their association with various animal and environmental factors had been investigated previously only on a small scale (112, pp. 39–40).

To address these unknowns, researchers designed a study that was billed as a ‘practical collaboration between veterinary practices which collect the data, and research workers who co-operate in designing the survey, providing the record forms, and analyzing the results’ (113, p. 26). It commenced with a survey completed by veterinary clinicians on every case of lameness they attended in 1977. As the only lameness expert on the research team, Weaver designed the template on which they recorded their observations. They were asked to indicate the anatomical seat of lameness on a standardized anatomical diagram, select from a long list of possible diagnoses, and answer questions about the cow, her feet, environment and habits (114). Due to the activities of the BVA’s Clinical Observation Unit (founded in 1962 on the model of the Royal College of General Practitioners’ Epidemiological Observation Unit), clinicians were, by now, accustomed to participating in surveys of this kind (115, 116). Weaver provided further encouragement in his capacity as an officer of the British Cattle Veterinary Association (BCVA, established in 1967 with Weaver as Honorary Secretary). One of the BCVA’s objectives was to advance knowledge pertaining to cattle. By the mid-1970s it had 230 members, of whom 130 worked in general practice (113, 117).

Built on the back of veterinary clinical labor, the lameness survey broke new ground in veterinary epidemiology, then a nascent discipline with very limited institutional presence (118). 9,130 reports of lameness were returned by 146 clinicians working in 48 practices. Pertaining to 7,528 cows in 1,823 herds that contained a total of 136,900 dairy cattle or 3 % of the national dairy herd, it was one the biggest surveys of a single health problem ever undertaken in Britain. IRAD statisticians, supported by the Computer Department of Rothamsted Experimental Station, turned these individual experiences of lameness into big data. They coded the survey returns, transferred entries onto punch cards for entry into the computer, and analyzed them using the Generalized Linear Interactive Modelling (GLIM) software programme (114).

The findings confirmed, refined and statistically codified the observations that clinicians had been making since the late 1950s about the types, causes and incidence of lameness in modern dairy cows (12). They confirmed that lameness mainly affected the foot, particularly the outer claw of the hind limb. The commonest causes were foul in the foot, white line abscess and sole ulcer. As herds grew larger, foul became less common and sole ulcer more common. Lameness incidence was now 5.5% among cows attended by veterinary surgeons, compared to 3.88% of all cows recorded by the 1957/8 national survey. Whereas *Lameness in Cattle* (1972) had estimated national losses at £2 m per annum, this survey pointed to £15.5 million in 1977/8 (114). Inclusion of lame cows treated only by farmers would have produced an even higher figure, as shown by a slightly later survey conducted by the University of Edinburgh Veterinary School, which estimated an annual incidence of 25%, costing more than £35 m (119, 120).

Detailed statistical analysis revealed the relative contributions of different factors and the interactions between them, including claw condition, cow origin (home bred cows were less vulnerable), girth (a proxy for bodyweight, with larger cows more frequently affected), age (younger cows were more prone to foul and laminitis, and older ones to sole erosions and white line disease), breed (Friesians and Shorthorns were more prone than the lighter Ayrshire, Guernsey and Jersey breeds), housing system (housed cows were more vulnerable than those at pasture, and incidence was higher in cubicles than straw yards), herd size (those with less than 50 or more than 200 cows were particularly badly affected), and stage of lactation (most problems occurred within 50 days of giving birth). Wet and dirty environments were implicated in foul; walking on hard surfaces in white line disease; and housing for long periods, lack of exercise and high body weight in sole erosion and ulceration. Wet weather had only a limited effect (121–123).

It took nearly 10 years for IRAD statisticians to produce these detailed findings. Meanwhile, its pathologists investigated the mechanisms through which variables operated, focusing particularly on the common, painful condition of sole ulcer. This was very difficult to treat. Many veterinary practices concocted their own topical remedies to try and promote regrowth of the horn (12). Pathologists looked at how the strength and wear of hoof horn was affected by physiology (nutrition, age, pregnancy, lactation), environment (water, slurry, urine and abrasive surfaces), heredity, and intercurrent disease. Supported by clinicians who had participated in the original survey, they also confirmed the effectiveness of footbaths in preventing lameness (124–126). (Significantly this was prior to the appearance of digital dermatitis (caused by Treponema bacteria), whose management is now the major reason for using footbaths).

IRAD researchers communicated their findings back to veterinary clinicians via presentations to the 1978 BVA congress (the first since 1960 to dedicate a panel to lameness) and BCVA meetings (127, 128), where clinicians continued to share and compare experiences such that ‘the real work and learning was done in the bar’ (12, p. 93). They wrote accessible, illustrated articles for clinicians that described different lameness lesions, their predisposing causes, diagnosis, treatment, and prevention through adjustments to breeding strategies, housing and day to day management (129, 130). Weaver continued to bridge these communities, incorporating research findings into checklists for veterinary clinicians to use in conducting organized, systematic, herd-level lameness enquiries (131).

One important observation drawn from the lameness survey was that veterinary practices varied significantly in the types of lameness lesions that they recorded. This may account for why, in their later studies, IRAD scientists preferred to use lameness data collected from IRAD’s own herds (132). Unable to fully explain these variations by reference to lameness biology, they concluded that personal differences in recording had played a role, and that consequently the veterinary practice was itself a variable that needed to be considered when modelling the effects of different factors on lameness (121). In other words, veterinary clinicians were unreliable observers. Here, we see the emergence of attitudes and knowledge hierarchies that would develop further under the impetus of EBVM. Despite their seminal contributions to IRAD’s lameness research programme, and the considerable knowledge of lameness they had generated in the 20 years prior to IRAD’s involvement, clinicians’ opinions on the problem were not scientifically trustworthy.

By 1987, surpluses in agricultural production, the cost of supporting farmers via the Common Agricultural Policy, and the imposition of milk quotas for dairy farmers, meant that research aimed at removing disease impediments to dairy productivity was no longer a government priority. Meanwhile concerns about the government’s scientific response to BSE raised questions about the structure of animal disease research. Research budgets were slashed and IRAD merged with three other institutions to form a new Institute for Animal Health. Payne departed as Director. This changing context led to the termination of the first substantial programme of British research into cattle lameness (37, 97, 133, 134).

## 4 Discussion

This article has broken new ground in veterinary history by presenting the first social historical account of dairy cow lameness in Britain. Using diverse veterinary publications from the period c.1930–87, it has traced the historical transformation of lameness from a low-profile, sporadic problem of locomotion that was understood particularly by reference to lameness in the horse, into one of the most significant endemic threats to bovine welfare, health and productivity (Table 1). This transformation was driven by biological changes to lameness pathology and epidemiology under the influence of new feeding, breeding and husbandry practices, and by social circumstances that shaped how, when, and by whom, knowledge of these changes was generated. Until the late 1970s, veterinary clinicians led the way. Working in private practice and veterinary school field stations, they translated individual encounters with lame cows into collective understandings that rendered visible its diverse pathologies, multi-factorial etiology, and economic and welfare implications. Veterinary scientists showed little interest prior to the 1977 launch of IRAD’s major research programme. Eschewing simplistic, moralistic attributions of ‘neglect’, this article has attempted to explain their attitudes. It has shown also that even after lameness research commenced, knowledge-building continued to be shaped by clinicians’ expertise and experience. Nevertheless, scientists came to regard them as unreliable observers, heralding a new hierarchy of knowledge that was to achieve full expression with the rise of EBVM.

Today, the role of veterinary clinicians in building knowledge of lameness is largely forgotten. In reconstructing and drawing attention to their contributions, this article complicates the conventional, linear model of knowledge production, in which science precedes and directs practice, and advisors like veterinary clinicians function as mere conduits of information from scientific experts to farming users (135, 136). It contributes to a broader historiography of medicine and science that calls for greater attention to practice-based knowledge production (137). It also introduces a historical dimension to contemporary explorations of the rise of EBVM (138) and the role of veterinary clinicians in generating field-based knowledge (139), which document clinicians’ situated understandings of disease and their distinction from epidemiological ways of knowing (140–142).

Taking the lead from recent social scientific studies, future research could explore the historical role of farmers in informing and shaping veterinary clinical knowledge (143). Collecting oral histories of lameness would enhance the evidence base used for this study. Extending the investigation forward in time would allow analysis of the impact of digital dermatitis. National comparisons would be illuminating. Most western countries experienced a similar ‘lameness transition’ as they moved to more intensive production practices. However, the importance of contextual factors in shaping veterinary responses in Britain suggests that the construction of lameness knowledge may have followed different trajectories in other nations, and that its investigation could be similarly fruitful in opening up wider perspectives on 20th century veterinary worlds. Further research is also required to determine whether the importance of veterinary knowledge-building in clinical settings was specific to lameness, or a more general feature of twentieth century British veterinary medicine. In broadening the focus of enquiry beyond lameness, such research would extend the efforts made by this study to demonstrate the historical significance of endemic health problems and their relationship to changing production practices.

## Data Availability

The original contributions presented in the study are included in the article/supplementary material, further inquiries can be directed to the corresponding author.
